# The Beneficial Changes on Inflammatory and Endothelial Biomarkers Induced by Metabolic Surgery Decreases the Carotid Intima-Media Thickness in Men

**DOI:** 10.3390/biom12121827

**Published:** 2022-12-07

**Authors:** Pilar Cobeta, Roberto Pariente, Alvaro Osorio, Marta Marchan, Luis Blázquez, David Pestaña, Julio Galindo, José I. Botella-Carretero

**Affiliations:** 1 Department of Anesthesiology, Hospital Universitario Ramón y Cajal, 28034 Madrid, Spain; 2 Instituto Ramón y Cajal de Investigación Sanitaria—IRyCIS, Hospital Universitario Ramón y Cajal, 28034 Madrid, Spain; 3 Department of Inmunology, Hospital Universitario Ramón y Cajal, 28034 Madrid, Spain; 4 Department of Angiology and Vascular Surgery, Hospital Universitario Ramón y Cajal, 28034 Madrid, Spain; 5 Department of Endocrinology and Nutrition, Hospital Universitario Ramón y Cajal, 28034 Madrid, Spain; 6 Department of General and Digestive Surgery, Hospital Universitario Ramón y Cajal, 28034 Madrid, Spain

**Keywords:** metabolic surgery, cardiovascular risk, carotid intima-media thickness, inflammation, testosterone

## Abstract

Obesity increases cardiovascular risk in men through several mechanisms. Among them, low-grade chronic inflammation and obesity-associated hypogonadism have been described. We aimed to study the effects of metabolic surgery on the carotid-intima media thickness through changes in inflammatory, endothelial biomarkers, and testosterone. We included 60 men; 20 submitted to laparoscopic Roux-en-Y gastric bypass (RYGB), 20 to sleeve gastrectomy (SG), and 20 to lifestyle modification (controls). Several inflammatory and endothelial biomarkers and total testosterone (TT) were measured at baseline and six months after surgery. Free testosterone (FT) was calculated, and carotid intima-media thickness (cIMT) was measured by ultrasonography. Compared to controls, cIMT decreased after surgery concomitantly with CRP, PAI-1, sICAM-1, and IL-18 (*p* < 0.01) and with an increase in sTWEAK (*p* = 0.027), with no differences between RYGB and SG. The increase in TT and FT after surgery correlated with the changes in cIMT (*p* = 0.010 and *p* = 0.038, respectively), but this association disappeared after multivariate analysis. Linear regression showed that sTWEAK (ß = −0.245, *p* = 0.039), PAI-1 (ß = 0.346, *p* = 0.005), and CRP (ß = 0.236, *p* = 0.049) were associated with the changes in cIMT (R^2^ = 0.267, F = 6.664, *p* = 0.001). In conclusion, both RYGB and SG induced improvements in inflammation and endothelial biomarkers that drove a decrease in cIMT compared to men with obesity who submitted to diet and exercise.

## 1. Introduction

Obesity is a global health problem with a high prevalence, and it is associated with major chronic diseases including cancer, cardiovascular diseases (CVD), and diabetes mellitus (DM) [1,2]. Surgical treatment of severe obesity is the most effective as it maintains long-term weight loss, improves and even resolves many of the comorbidities associated with obesity, reduces cardiovascular risk, and increases patients’ survival [3,4,5].

Among the many cardiovascular risk markers commonly employed, an increase in the common carotid intima-media thickness (cIMT) has been associated with unfavorable classical cardiovascular risk factors and systemic atherosclerosis [6]. Therefore, non-invasive assessment of cIMT by high-resolution B-mode ultrasonography is widely used as a predictor of future stroke and myocardial infarction, and it can be considered a surrogate clinical endpoint [7]. Metabolic surgery has been shown to diminish cIMT in some previous studies, as reported by a meta-analysis [8]. We have also recently reported that both Roux-en-Y gastric bypass (RYGB) and sleeve gastrectomy (SG) induce a similar decrease in cIMT superior to lifestyle modification in both women and men with obesity and high cardiovascular risk [9,10].

Different mechanisms have been proposed to explain the link between obesity and atherogenesis, and both inflammation and insulin resistance play a well-recognized role in this process [11,12]. The endothelial surface expresses adhesion molecules, which recruit inflammatory cells propagating inflammation in the vessel wall and promoting the development of atherosclerotic plaque [13]. In patient candidates for metabolic surgery, pro-inflammatory and adiposity biomarkers have been shown to be associated with progressive subclinical atherogenesis measured by cIMT [14]. 

Further, the beneficial effects of RYGB on markers of atherosclerosis such as brachial artery flow-mediated dilation and cIMT paralleled a decrease in C-reactive protein (CRP) [15]. In addition, after SG, the reduction in cIMT was associated with a decrease in insulin resistance and several pro-inflammatory markers [16]. Published data of our own have also shown this association between the decrease in inflammatory markers after both SG and RYGB and the reduction in cIMT in women [17]. Proteomic approaches demonstrated specific molecular mechanisms, genes, and proteins that improve adipose tissue function after metabolic surgery, characterized by increased glucose uptake, higher insulin sensitivity, and lower inflammation [18]. 

In men, circulating testosterone may also play a role in atherogenesis. Male hypogonadism is associated with dyslipidemia, atherosclerosis, CVD, and DM, and testosterone supplementation therapy in men has been shown to improve the lipid profile, glycemia, and insulin resistance [19,20]. However, whether testosterone replacement therapy is beneficial for cardiovascular outcomes is still debated [21]. Regarding men who submitted to metabolic surgery, more than half may present secondary hypogonadism, and its resolution can be achieved in most of them after surgery [22]. We have previously shown that the decrease in cIMT correlated with the increase in total testosterone and weight loss after metabolic surgery in men [10,23]. 

In this study, we aimed to evaluate the changes in several circulating inflammatory and endothelial biomarkers in men after RYGB and SG and their association with the changes in cIMT, directly comparing both surgical techniques in a prospective study. We also aimed to characterize its association with changes in circulating testosterone.

## 2. Materials and Methods

### 2.1. Patients and Study Design

Sixty men with severe obesity and high cardiovascular risk defined by the presence of metabolic syndrome (according to the American Heart Association and National Heart, Lung, and Blood Institute) were included in this study. The patients were all of Caucasian origin.

Of them, 20 were submitted for laparoscopic RYGB and 20 for SG. The other 20 patients were treated with diet and lifestyle modifications. This was not a randomized study, and patients were allocated to each surgical technique in accordance with the international guidelines for metabolic surgery and our hospital’s protocol. Our current protocol allocates patients with more obesity-associated complications preferentially to RYGB. 

Exclusion criteria included mental impairment, an uncontrolled psychiatric condition or active substance abuse, active neoplastic disease, and unstable or incurable serious pre-existing comorbidities. Patients were evaluated at baseline and 6 months after surgery or after starting conventional treatment with diet and lifestyle modification, respectively.

This study was conducted between 2018 and 2019 in accordance with the Declaration of Helsinki and approved by the Institutional Review Board (or Ethics Committee) of Hospital Universitario Ramón y Cajal (code: PI18/00132). Written informed consent was obtained from all subjects involved in this study.

### 2.2. Procedures

Between 8 AM and 9 AM, and after a 12-h overnight fast, an indwelling intravenous line was placed in a forearm vein, and after 15–30 min, basal blood samples were obtained in each patient and immediately centrifuged at a cold temperature and then stored at −80 °C until further assayed. 

Office blood pressure and anthropometric parameters were also recorded, and body mass index (BMI) was calculated as weight in kg divided by height in square meters. Excess body weight (EBW) was calculated as the difference between baseline body weight and the weight corresponding to a BMI of 25 kg/m^2^.

The main characteristics of the RYGB procedure include a 20–40 mL gastric pouch, a biliopancreatic limb measuring 80–100 cm from the Treitz ligament, and a 120–200 cm-long alimentary limb. SG was performed with a laparoscopic linear stapler calibrated with a 36F orogastric tube. There were no major postoperative complications, and all the patients could be discharged from the hospital in the two to five days following surgery.

cIMT was estimated by ultrasonography at baseline and six months after the intervention. Ultrasonography was performed in all subjects by the same experienced vascular surgeon (A.O.) using a Toshiba Nemio (Toshiba Corporation, Otawara-Shi, Japan) with a linear multifrequency 8–12 MHz ultrasound probe. The vascular surgeon was blinded to the treatment applied, including the surgical technique. cIMT was measured as the distance between the intima-span layer and the media-adventitia layer in both carotid arteries at the far wall of the common carotid artery, 1 cm proximal to the carotid bifurcation. Measurements were obtained at maximum B-mode sonography resolution during diastole. Six measurements were conducted at each carotid artery, and their median value was assigned to each vessel. Then the arithmetical mean of the two arteries was used for the analysis. The intra-observer coefficient of variation for the cIMT measurement was 9.7% for the investigator who performed the ultrasonography. Data on the change in cIMT of these patients were reported before [10].

### 2.3. Assays

Circulating inflammatory and endothelial biomarkers were analyzed in duplicate in serum or plasma samples by commercial enzyme-linked immunosorbent assays (ELISA): Human plasminogen activator inhibitor 1 (PAI-1) ELISA kit, human soluble intercellular adhesion molecule 1 (sICAM-1) ELISA kit, human soluble vascular cell adhesion protein 1 (sVCAM-1) ELISA kit, human interleukin 6 (IL-6) high-sensitivity ELISA kit, human interleukin 18 (IL-18) ELISA kit, and human soluble tumor necrosis factor-like weak inducer of apoptosis (sTWEAK) instant ELISA kit (Thermofisher Scientific, Bender MedSystems GmbH, Campus Vienna Biocenter 2, Vienna, Austria). CRP was measured by a high-sensitivity colorimetric method (Multigen CRP Vario assay, Sentinel CH, Milan, Italy). All these measurements had a coefficient of variation < 10%.

Assays and reference ranges for TT and sex hormone binding globulin (SHBG) were previously reported [10]. Fasting insulin was measured by immunochemoluminescence (Immulite 2000, Siemens Healthcare Diagnostics Inc., Gwynedd, UK), with a CV < 10%. Insulin resistance in the fasting state was estimated by homeostasis model assessment (HOMA-IR). Levels of HDL cholesterol were measured in the supernatant after plasma precipitation (Boehringer Mannheim GmbH, Mannheim, Germany). Levels of total cholesterol and triglycerides were measured by enzymatic methods (Menarini Diagnostica, Florence, Italy). The LDL cholesterol level was calculated using Friedewald’s formula.

### 2.4. Sample Size

A priori sample size analysis for the changes in cIMT was performed as reported before [10]. For the changes in circulating inflammatory markers, a total sample size of 18 subjects was enough to detect a mean difference of 2.5 mg/dL with an SD of 3.5 for CRP, 12 subjects to detect a mean difference of 45 pg/mL with an SD of 55 for IL-18, and 17 subjects to detect a mean difference of 45 pg/mL with an SD of 65 for PAI-1 in the follow-up period with 1 − β = 0.80 and α = 0.05. Analyses were performed using the GRANMO 7.12 online tool (https://www.imim.es/ofertadeserveis/software-public/granmo/index.html accessed on 1 February 2018).

### 2.5. Statistical Workout

Results are expressed as means ± SD unless otherwise stated. The Kolmogorov–Smirnov statistic was applied to continuous variables. Logarithmic or square root transformations were applied as needed to ensure the normal distribution of the variables. A one-way analysis of variance followed by Tukey tests was used to compare the central tendencies of the different groups. Kruskal–Wallis test followed by Wilcoxon tests was used in the case of non-normal distributed variables. To evaluate discontinuous variables, we used the χ2 test and Fisher’s exact test as appropriate. 

Comparisons of continuous variables before and after bariatric surgery were performed using repeated measures GLM analysis, and the group of subjects (controls, SG, and RYGB) was introduced as the between-subject effect. Bivariate correlation was employed to study the association between two continuous variables using Pearson or Spearman’s tests as appropriate. Multiple linear regression (probability of F to enter ≤ 0.05, probability of F to remove ≥ 0.1) was applied to find the effects of several dependent variables and their interactions on the decrease in cIMT and other independent variables. Analyses were performed using SPSS 18 (SPSS Inc., Chicago, IL, USA). *p* < 0.05 was considered statistically significant.

## 3. Results

### 3.1. Baseline Characteristics

Sixty patients with an age of 48 ± 9 y (controls *n* = 20, age 48 ± 8 y, SG *n* = 20, age 46 ± 9 y, RYGB *n* = 20, age 51 ± 9 y, *p* = 0.190) were evaluated at baseline and six months after surgery (Table 1). At baseline, those submitted to surgery had slightly lower fasting insulin and insulin resistance compared with controls. In addition, those patients who submitted to RYGB showed lower concentrations of LDL cholesterol and a higher proportion of hypertension. They also needed more medications (18 on antihypertension drugs, 12 on statins, and 8 on oral antidiabetics vs. SG with 7 on antihypertension drugs, 6 on statins, and 6 on oral antidiabetics, and controls with 8 on anti-hypertension drugs, 6 on statins, and 7 on oral antidiabetics). Previous known cardiovascular events in the included patients were strokes (*n* = 1 in the diet group, *n* = 2 in the SG group) and isquemic heart diseases (*n* = 1 in the RYGB group). The lack of randomization might explain these differences.

### 3.2. Effects of Bariatric Surgery on Anthropometric, Biochemical Variables and cIMT

As expected, total weight loss, BMI, EWL, blood pressure, fasting glucose, and insulin decreased after both RYGB and SG compared with controls, with no differences between these two surgical techniques. HDL cholesterol increased after both RYGB and SG compared with controls, with no differences between types of surgery (Table 2). The cIMT decreased in patients after surgery regardless of the technique used but did not change in controls submitted to conventional treatment (Table 2).

### 3.3. Effects of Bariatric Surgery on Inflammation and Endothelial Biomarkers 

**Figure 1:**
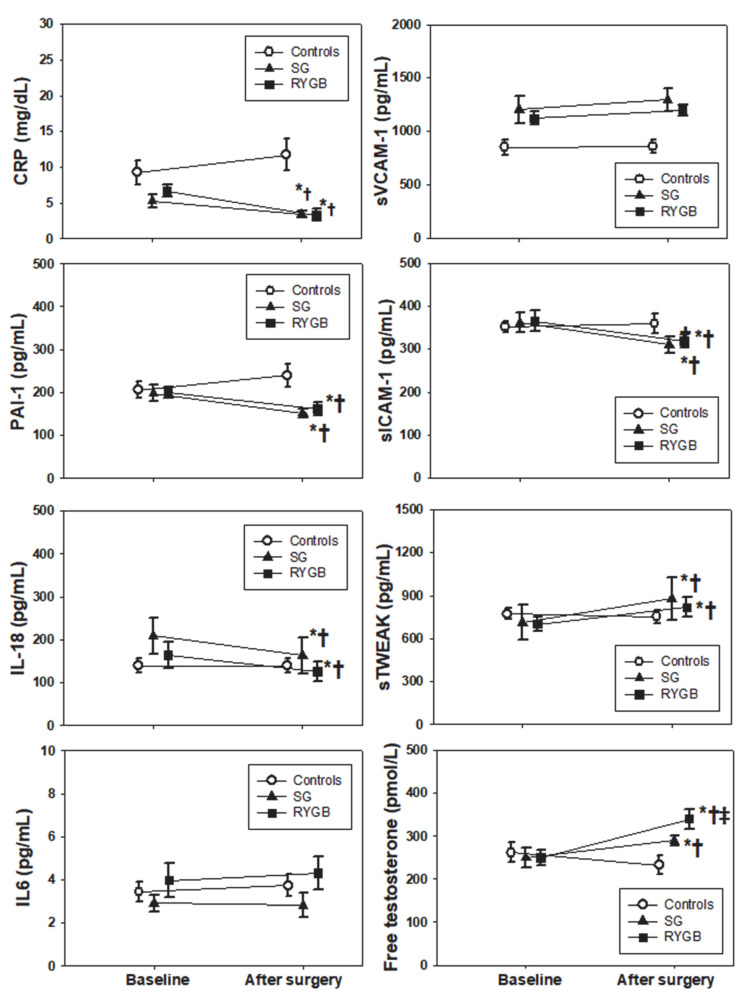
Changes in circulating inflammatory and endothelial biomarkers in the included men after metabolic surgery. Symbols represent means, and error bars represent SEMs. * *p* < 0.05 from baseline, † *p* < 0.05 vs. controls, ‡ *p* < 0.05 vs. SG.

Changes in inflammatory and endothelial biomarkers after metabolic surgery showed a beneficial pattern (Figure 1). CRP decreased after both RYGB and SG compared to controls (*p* = 0.001), with no difference between these surgical techniques (*p* = 0.204). Similar findings were observed for the decrease in PAI-1, sICAM-1, and IL-18 (*p* < 0.01 vs. controls and *p* > 0.05 between surgical techniques), whereas no change was observed for sVCAM-1 or IL-6 (*p* > 0.05 in all comparisons). The increase in sTWEAK after surgery also indicated an amelioration of inflammation, with no differences between SG and RYGB (*p* = 0.027 and *p* = 0.89, respectively). As expected, both TT and FT showed an increase after surgery but not in controls (*p* < 0.01), and this increment was higher after RYGB (Figure 1).

### 3.4. Ancyllary and Multivariate Analyses 

When all participants were pooled together (both men submitted to surgery and those to diet and exercise), the observed changes in cIMT at follow-up showed positive correlations with the changes in CRP and PAI-1. Circulating sTWEAK showed a near-significant bivariate negative correlation with the changes in cIMT. Circulating TT and FT were inversely correlated with the changes in cIMT, CRP, and IL-18 and positively correlated with sTWEAK (Table 3).

A multivariate linear regression was performed, introducing the change in cIMT as the dependent variable and changes in CRP, PAI-1, sICAM-1, sVCAM-1, IL-6, IL-18, sTWEAK, and FT (total testosterone and SHBG were not introduced to avoid collinearity) as independent variables. The model showed that an increase in sTWEAK (ß = −0.245, *p* = 0.039) and a decrease in PAI-1 (ß = 0.346, *p* = 0.005) and CRP (ß = 0.236, *p* = 0.049) were associated with the decrease in cIMT (R^2^ = 0.267, F = 6.664, *p* = 0.001) after metabolic surgery in men. Another regression model that included age as a covariate did not change these results significantly, and age was not associated with changes in cIMT (ß = 0.116, *p* = 0.351).

## 4. Discussion

Our results show that in men with obesity, both RYGB and SG induce an improvement in inflammation and endothelial biomarkers that drives a decrease in cIMT compared to obese men submitted to diet and exercise. Although circulating testosterone was inversely correlated with this decrease in cIMT, multivariate analysis did not confirm this finding. 

The association of low circulating testosterone in males with cardiovascular disease and cIMT has not been consistently demonstrated in previous studies. One study showed that cIMT was negatively correlated with TT in 115 men younger than 70 years without a history of cardiovascular events [24], but other studies failed to show an association of circulating androgens with cIMT [25,26]. We have recently shown that those men with hypogonadism who achieve a complete normalization of testosterone after metabolic surgery experience a significant decrease in cIMT compared to those who remain hypogonadal [23]. It seems that the increase in circulating testosterone after metabolic surgery is the result of weight loss and the amelioration of insulin and leptin resistance [27,28]. This in turn produces a concomitant increase in kisspeptin concentration that restores the hypothalamic–pituitary–gonadal axis [27] with the normalization of testosterone. However, this may have only a mild effect on the decrease in cardiovascular risk as measured by the cIMT, not as powerful as the one induced by the amelioration of inflammatory and endothelial biomarkers, which may explain why this effect of testosterone disappeared after multivariate analysis in our study.

Chronic low-grade inflammation plays a well-recognized role as a link between obesity and cardiovascular disease [11,12] and cIMT is a strong predictor of the latter [6]. Although the exact mechanism of this low-grade inflammation is not fully understood, there is clear evidence that infiltrating immune cells in adipose tissue, mainly macrophages, play a significant role in this process [29]. Physiologically, immune cells from both main lines, myeloid and lymphoid, contribute to tissue repair and apoptosis of damaged or infected cells with the production of different cytokines and other pro-inflammatory factors such as free radicals, nitric oxide, and others [30]. With adipose tissue expansion and its dysregulation, together with the occurrence of the ectopic fat depot, an increased circulating concentration of inflammatory cytokines occurs, and this can potentially modulate atheroma development. Endothelium expresses adhesion molecules, such as ICAM and VCAM, which recruit inflammatory cells, including monocytes and lymphocytes, and can further propagate inflammation in the vessel wall. This eventually promotes the development of the atherosclerotic lesion by inducing a proliferation of vascular smooth muscle cells and the secretion of pro-coagulant factors such as PAI-1 [13]. Therefore, we hypothesized that the observed decrease in cIMT after metabolic surgery in men could be mainly associated with an improvement of inflammation and endothelial biomarkers. Despite many studies showing in the past a decrease in several inflammatory markers after metabolic surgery [15,31,32], to our best knowledge, ours is the first one that explores this association with cIMT and circulating androgens in a prospective design in men, directly comparing the effects of RYGB with SG. 

Among the different inflammatory markers analyzed in the present study, IL-18 has been associated with increased cardiovascular risk [33], obesity [34,35], and insulin resistance [36]. It can be secreted in adipose tissue [37], up-regulated by other inflammatory cytokines [38], and able to stimulate the production of both tumor necrosis factor-α and IL-6, which in turn can induce the production of CRP in the liver [39]. IL-6 may cause the progression of atherosclerosis by inducing endothelial dysfunction and lipoprotein oxidation, and it is an independent risk factor for coronary artery disease [30]. CRP is also associated with a higher risk of cardiovascular disease, as individuals with obesity and higher CRP show higher coronary artery calcium scores and increased cIMT [40]. All these cytokines have been shown to decrease after metabolic surgery [15,31,32,41] and to be associated with a postsurgical decrease in cIMT [14,42]. In our present study, we have been able to confirm a decrease in IL-18 and CRP after both RYGB and SG, but multivariate analysis retained only CRP together with sTWEAK and PAI-1 as those variables associated with the observed changes in cIMT.

TWEAK is a cytokine belonging to the tumor necrosis factor superfamily that induces a high number of physiological and pathological processes depending on cell type through its receptor fibroblast growth factor inducible 14 (Fn14) [43]. Both TWEAK and Fn14 are expressed in the arterial wall, both in healthy arteries and atherosclerotic plaques [44], and participate in different stages of atherosclerotic plaque development [45]. sTWEAK is a 156-aa proteolytic product derived from the full-length, membrane-bound TWEAK and binds the Fn14 receptor [46]. Under pathological conditions such as atherosclerosis, arterial Fn14 expression is upregulated, thus favoring sTWEAK binding and retention in the pathological tissues and decreasing its circulating levels. Therefore, low levels of sTWEAK are found in different atherosclerotic states such as stable coronary artery disease, chronic kidney disease, or peripheral artery disease [47]. In our study, the increase in sTWEAK after both RYGB and SG compared to controls was associated, after the multivariate analysis, with the decrease in cIMT in men, in agreement with previously reported data from our own cohort of women submitted to metabolic surgery [17].

PAI-1 is a member of the serine protease inhibitor superfamily that regulates the fibrinolytic system through inhibition of tissue plasminogen activator and urokinase-type plasminogen activator, and it is considered the main inhibitor of fibrinolysis [48]. Elevated plasma levels of PAI-1 have been associated with obesity, metabolic syndrome, and cardiovascular disease. PAI-1 is produced by a variety of cells contained in adipose tissue: pre-adipocytes, mature adipocytes, stromal cells, endothelial cells, smooth muscle cells, and macrophages [49]; therefore, PAI-1 levels directly correlate with cIMT both in young and adult obese subjects [50]. Moreover, high circulating concentrations of PAI-1, together with insulin resistance and the presence of the metabolic syndrome, predicted higher cIMT and/or carotid atherosclerotic plaque [51]. We previously reported a decrease in PAI-1 after metabolic surgery in women but failed to demonstrate a correlation with the decrease in cIMT [17]. Conversely, our present data show that in men, PAI-1 decreases after both RYGB and SG, and it is associated with the decrease in cIMT after surgery. 

### Limitations of the Study and Generalization

First, our present study has the major limitation of a lack of randomization in the allocation of the patients to the different groups of interventions. Second, although the effect of testosterone on cIMT was significant in bivariate correlations, it disappeared after multivariate analysis in our study, possibly reflecting the small sample size for this outcome as the sample size calculations were performed to detect differences in cIMT and inflammatory biomarkers. In addition, the generalizability of the results is limited because we evaluated the patients after a short-term period of 6 months, so the long-term beneficial effects of metabolic surgery on cIMT and cardiovascular events could not be evaluated.

## 5. Conclusions

Both RYGB and SG induce an improvement of inflammation and endothelial biomarkers that drive a decrease in cIMT compared to men with obesity who submit to diet and exercise. Although circulating testosterone was inversely correlated with this decrease in cIMT, multivariate analysis did not confirm this finding, possibly reflecting a smaller effect compared to the inflammatory and endothelial biomarkers. Future studies, preferably randomized and in the long term, are needed to confirm our results.

## Figures and Tables

**Table 1:** Baseline anthropometric and metabolic characteristics of the men included in this study.

	Controls (*n* = 20)	SG (*n* = 20)	RYGB (*n* = 20)
BMI (kg/m^2^)	44.0 ± 5.4	45.0 ±6.9	43.7 ± 7.2
EBW (kg)	59.4 ± 17.4	61.6 ± 21.2	56.7 ± 20.8
Hypertension (%)	10 (50)	10 (50)	18 (90) †‡
Type 2 DM (%)	8 (40)	6 (30)	10 (50)
cIMT (mm)	0.66 ± 0.10	0.65 ± 0.11	0.66 ± 0.13
Systolic BP (mmHg)	145 ± 16	141 ± 17	144 ± 16
Diastolic BP (mmHg)	87 ± 9	85 ± 11	87 ± 11
LDL (mmol/L)	3.0 ± 0.8	3.0 ± 0.9	2.1 ± 0.8 †‡
HDL (mmol/L)	1.1 ± 0.4	1.0 ± 0.2	1.0 ± 0.2
Glucose (mmol/L)	6.6 ± 2.3	5.9 ± 1.9	7.2 ± 3.3
Insulin (mU/L)	30 ± 16	19 ± 9 †	21 ± 14 †
HOMA-IR	9.5 ± 8.0	5.1 ± 3.1 †	6.4 ± 5.7 †

SG: sleeve gastrectomy, RYGB: Roux-en-Y gastric bypass, BMI: body mass index, EBW: excess body weight, DM: diabetes mellitus, cIMT: carotid intima-media thickness, BP: blood pressure, LDL: low-density lipoprotein cholesterol, HDL: high-density lipoprotein cholesterol, HOMA-IR: insulin resistance calculated by the homeostatic model assessment. † *p* < 0.05 vs. controls, ‡ *p* < 0.05 vs. SG.

**Table 2:** Six-month follow-up anthropometric and metabolic characteristics of the men included in this study.

	Controls (*n* = 20)	SG (*n* = 20)	RYGB (*n* = 20)
BMI (kg/m^2^)	45.2 ±7.1	33.2 ± 4.1 *†	31.6 ± 6.5 *†
EBW (kg)	3.7 ± 14.1	57.4 ± 18.2 *†	69.4 ± 26.2 *†
Hypertension (%)	10 (50)	1 (0.5) *	7 (35) *‡
Type 2 DM (%)	8 (40)	4 (20) *	2 (10) *
cIMT (mm)	0.67 ± 0.11	0.60 ± 0.09 *†	0.60 ± 0.12 *†
Systolic BP (mmHg)	151 ± 12	130 ± 12 *†	129 ± 17 *†
Diastolic BP (mmHg)	89 ± 8	84 ± 8 †	76 ± 12 *†
LDL (mmol/L)	3.2 ± 0.6	2.9 ± 0.8	1.9 ± 0.7
HDL (mmol/L)	1.0 ± 0.3	1.2 ± 0.2 *†	1.2 ± 0.4 *†
Glucose (mmol/L)	7.0 ± 2.9	5.4 ± 0.9 †	5.5 ± 1.7 *†
Insulin (mU/L)	27 ± 14	10 ± 6 *†	7 ± 3 *†
HOMA-IR	15.2 ± 28.6	2.4 ± 1.6	3.2 ± 5.8

SG: sleeve gastrectomy, RYGB: Roux-en-Y gastric bypass, BMI: body mass index, EBW: excess body weight, DM: diabetes mellitus, cIMT: carotid intima-media thickness, BP: blood pressure, LDL: low-density lipoprotein cholesterol, HDL: high-density lipoprotein cholesterol, HOMA-IR: insulin resistance calculated by the homeostatic model assessment. * *p* < 0.05 from baseline, † *p* < 0.05 vs. controls, ‡ *p* < 0.05 vs. SG.

**Table 3:** Bivariate correlation of changes in inflammatory and endothelial biomarkers with cIMT and testosterone.

	Δ cIMT		Δ TT		Δ FT	
	r	*p*	r	*p*	r	*p*
Δ CRP	0.317	0.014	−0.444	<0.001	−0.259	0.046
Δ PAI-1	0.381	0.030	−0.238	0.067	−0.145	0.268
Δ sICAM-1	0.080	0.544	−0.097	0.459	−0.051	0.698
Δ sVCAM-1	0.045	0.736	−0.189	0.149	−0.014	0.914
Δ IL-6	0.156	0.234	−0.024	0.855	−0.105	0.424
Δ IL-18	0.261	0.056	−0.333	0.014	−0.271	0.048
Δ sTWEAK	−0.254	0.050	0.303	0.019	0.299	0.020
Δ TT	−0.428	0.010	1.000	-	0.849	<0.001
Δ FT	−0.269	0.038	0.849	<0.001	1.000	-

cIMT: carotid intima-media thickness, TT: total testosterone, FT: free testosterone, PAI-1: plasminogen activator inhibitor 1, sICAM-1: soluble intracellular adhesion molecule 1, sVCAM-1: soluble vascular adhesion molecule 1, IL: interleukin, sTWEAK: soluble tumor necrosis factor-like weak inducer of apoptosis.

## Data Availability

Restrictions apply to the availability of the data generated or analyzed during this study to preserve patient confidentiality or because they were used under license. The corresponding author will, on request, detail the restrictions and any conditions under which access to some data may be provided.
